# Progressive supranuclear palsy and corticobasal degeneration: novel clinical concepts and advances in biomarkers

**DOI:** 10.1590/0004-282X-ANP-2022-S134

**Published:** 2022-08-12

**Authors:** Jacy Bezerra Parmera, Marcos Castello Barbosa de Oliveira, Roberta Diehl Rodrigues, Artur Martins Coutinho

**Affiliations:** 1 Universidade de São Paulo, Faculdade de Medicina, Hospital das Clínicas, Departamento de Neurologia, São Paulo, SP, Brazil.; 2 Universidade de São Paulo, Faculdade de Medicina, Hospital das Clínicas, Instituto de Radiologia, Centro de Medicina Nuclear, Laboratório de Medicina Nuclear (LIM 43), São Paulo, SP, Brazil.; 3 Universidade de São Paulo, Faculdade de Medicina, Departamento de Radiologia, Laboratório de Medicina Nuclear (LIM 44), São Paulo, SP, Brazil.

**Keywords:** Parkinsonian Disorders, Tauopathies, Supranuclear Palsy, Progressive, Positron-Emission Tomography, Magnetic Resonance Imaging, Biomarkers, Transtornos Parkinsonianos, Tauopatias, Paralisia Supranuclear Progressiva, Tomografia por Emissão de Pósitrons, Imageamento por Ressonância Magnética, Biomarcadores

## Abstract

**Background:**

Progressive supranuclear palsy (PSP) and corticobasal degeneration (CBD) are sporadic adult-onset primary tauopathies clinically classified among the atypical parkinsonian syndromes. They are intrinsically related with regard to their clinical features, pathology, biochemistry, and genetic risk factors.

**Objectives:**

This review highlights the current knowledge on PSP and CBD, focusing on evolving clinical concepts, new diagnostic criteria, and advances in biomarkers.

**Methods:**

We performed a non-systematic literature review through the PubMed database. The search was restricted to articles written in English, published from 1964 to date.

**Results:**

Clinicopathologic and *in vivo* biomarkers studies have broadened PSP and CBD clinical phenotypes. They are now recognized as a range of motor and behavioral syndromes associated with underlying 4R-tauopathy neuropathology. The Movement Disorders Society PSP diagnostic criteria included clinical variants apart from the classical description, increasing diagnostic sensitivity. Meanwhile, imaging biomarkers have explored the complexity of symptoms and pathological processes related to corticobasal syndrome and CBD.

**Conclusions:**

In recent years, several prospective or clinicopathologic studies have assessed clinical, radiological, and fluid biomarkers that have helped us gain a better understanding of the complexity of the 4R-tauopathies, mainly PSP and CBD.

## RESUMO

**Antecedentes:**

A paralisia supranuclear progressiva (PSP) e a degeneração corticobasal (DCB) são taupatias esporádicas primárias clinicamente classificadas no grupo das síndromes parkinsonianas atípicas. Ambas estão intrinsecamente relacionadas no que concerne aos aspectos clínicos, patológicos, bioquímicos e genéticos.

**Objetivos:**

Abordar os avanços recentes no conhecimento da PSP e DCB, focando na evolução dos conceitos clínicos, critérios diagnósticos, e avanços em biomarcadores.

**Métodos:**

Trata-se de uma revisão não-sistemática através da base de dados PubMed. Foram revisados artigos escritos em língua inglesa, publicados desde 1964 até a presente data.

**Resultados:**

Estudos com biomarcadores e análises *postmortem* ampliaram os fenótipos conhecidos da PSP e DCB. Tais doenças englobam um espectro amplo de síndromes motoras e cognitivas, associadas a neuropatologia tau com isoforma predominante 4R. O atual critério para PSP estabeleceu novas variantes clínicas para além da descrição clássica, aumentando a sensibilidade diagnóstica. Concomitantemente, biomarcadores *in vivo* exploraram a complexidade dos sintomas e processos patológicos relacionados à síndrome e degeneração corticobasal.

**Conclusões:**

Recentemente, alguns estudos prospectivos e clinicopatológicos investigaram aspectos clínicos, radiológicos e de biofluidos, que ajudaram a melhor compreender a heterogeneidade e complexidade clínica das taupatias 4R, sobretudo PSP e DCB.

## INTRODUCTION

Progressive supranuclear palsy (PSP) and corticobasal degeneration (CBD) are sporadic neurodegenerative diseases clinically classified as atypical parkinsonian syndromes. After the development of levodopa to treat Parkinson’s disease (PD) in the late 1960s, diseases presenting with parkinsonism demonstrated to be wider than previously thought, and the group who had no improvement with levodopa treatment was then classified as atypical parkinsonism. 

PSP is a term coined in 1964 by Steele, Richardson, and Olszewski^1^ to describe a progressive disease involving the basal ganglia, cerebellum, and the brainstem with neuronal loss in the *substantia nigra*, in which patients presented parkinsonism, supranuclear gaze palsy, axial rigidity, pseudobulbar state and dementia. Currently, the term PSP is used for a specifically defined neuropathology, whereas the clinical syndrome classically depicted is now referred to as “Richardson’s syndrome” (PSP-RS). 

Corticobasal syndrome (CBS) and CBD were first described as a unique clinicopathological entity in 1967 and 1968 by Rebeiz et al.^2^ ^,^ ^3^, who reported clinical and pathological findings of three patients with progressive stiffness and awkward limb movements, dystonic posturing, and gait disorder. They identified asymmetrical frontoparietal cortical atrophy, loss of neurons in the *substantia nigra*, and swelling of the neuronal cell bodies with achromatic cells, and called this entity “corticodentatonigral degeneration with neuronal achromasia”^2^ ^,^ ^3^. Decades after the first description, the term “corticobasal degeneration” was coined by Gibb and Marsden in 1989^4^. Over time, it has become clear that the clinical features described by Rebeiz et al. were associated with various underlying pathologies^5^ ^-^ ^7^. Thus, Boeve et al.^8^ introduced the term “corticobasal syndrome” to embrace the constellation of symptoms leading to the initially described clinical phenotype, while CBD currently denotes the pathological disorder. 

PSP and CBD are conditions with notable overlap regarding their clinical presentation, pathological mechanisms, biochemistry, and genetic risk factors^9^. They are both characterized by the deposition of abnormal forms of tau protein, more specifically with the predominance of the four-repeat (4R) tau isoform and therefore classified as 4R-tauopathies. 

Furthermore, PSP and CBD often pose challenges in clinical practice, primarily because patients with typical signs or symptoms might have different pathologies, and patients with the same pathology can display diverse clinical syndromes. However, although predicting underlying pathology is difficult, proper investigation of clinical features remains essential and informative to understand their progression and pathological processes. Despite being considered ‘prototype’ primary tauopathies and possibly ideal for studying neuroprotective agents, the 4R-tauopathies are still severe and untreatable diseases with no validated biomarkers^10^. 

This review aims to provide an overview of the current knowledge on PSP and CBD, mainly covering the following topics: evolving clinical concepts, new diagnostic criteria, pathology aspects, and advances in biomarkers. 

## SEARCH STRATEGY

In this review, we performed a non-systematic literature search restricted to articles written in English based on PubMed literature from 1964 to date, using the terms “progressive supranuclear palsy”, “corticobasal degeneration”, “corticobasal syndrome”, and “four-repeat tauopathies”. Research papers with pathologically confirmed diagnoses were preferentially sought.

## NOVEL CONCEPTS: CLINICAL FEATURES AND NEW VARIANTS

PSP and CBD manifest with a broad and overlapping spectrum of clinical syndromes since the clinical presentation reflects the topographic distribution of histopathology more precisely than the specific underlying pathology. Initially considered atypical parkinsonian syndromes with mainly motor symptoms, it is recognized that they encompass a range of clinical phenotypes from movement, cognitive, and language abnormalities. PSP and CBD have a typical onset in the fifth to seventh decades and an average disease duration of 7.9 and 6.8 years, respectivelly^11^. 

PSP is the most common form of atypical parkinsonism, yet a rare disease, with an estimated prevalence of about 5-7 cases per 100.000 people^12^. However, other studies with autopsy series or different phenotypes beyond PSP-RS have found a higher prevalence of 18 cases per 100.000^13^ ^,^ ^14^. Also, little is known about the epidemiology of CBD and CBS. As CBD is a rare disease with various clinical phenotypes, accurate prevalence studies are lacking. A previous study showed an estimated CBD prevalence of 4.9-7.3 cases per 100.000 individuals^15^. Regarding CBS, a community-based Japanese study found a prevalence rate of 6 per 100.000^16^, while a Russian study showed an age-standardized incidence rate of 0.02 cases per 100.000 individuals^17^. 

Following hypothetical models from other neurodegenerative diseases, PSP is currently considered a clinicopathological continuum from a presymptomatic phase through a suggestive-of-PSP phase, in which individuals do not fulfill Movement Disorders Society (MDS) PSP criteria but present mild PSP symptoms, later evolving to a known clinical variant^18^. 

In the first two years of presentation, approximately two-thirds of patients with PSP pathology present different clinical variants and only years later progress to PSP-Richardson syndrome^19^. PSP clinical variants are presently named according to their predominant clinical features and comprise seven phenotypes.

The classic PSP clinical presentation is currently referred to as Richardson syndrome (PSP-RS), presenting as a levodopa-resistant, axial-predominant, symmetrical akinetic-rigid parkinsonism with early postural instability and frequent falls and slow vertical saccades lately evolving to vertical supranuclear gaze palsy. Subtle personality changes are frequently observed, including apathy and disinhibition, cognitive executive dysfunction, pseudobulbar state, dysarthria, dysphagia, and cervical dystonia^9^. Notably, vertical supranuclear gaze palsy might not be present until three or four years of disease onset. In contrast, other oculomotor dysfunctions such as absent vertical optokinetic nystagmus, square wave jerks, decreased vertical saccades velocity, and apraxia of eyelid opening usually are presented earlier^18^. PSP-RS probably accounts for 24-50% of PSP cases^10^ ^,^ ^20^. 

PSP-parkinsonism (PSP-P) is the second most common phenotype and the most difficult to distinguish from Parkinson’s disease. Individuals with a PSP-P clinical variant often present asymmetrical or symmetrical bradykinesia, tremor, and rigidity, which initially may be responsive to levodopa and have a slower progression rate than PSP-RS patients^11^. 

The PSP with progressive gait freezing (PSP-PGF) variant, previously named pure akinesia with gait freezing (PAGF), is a rare clinical phenotype characterized by an isolated gait disorder without other PSP classical features. There is a progressive gait disturbance with freezing of gait and starting hesitation. Worth mentioning, this clinical variant is considered highly predictive of PSP underlying pathology^21^.

The MDS PSP criteria also recognize clinical phenotypes that initially are mainly cognitive syndromes. One of these is the PSP-speech and language variant (PSP-SL), whose main symptoms are agrammatism and apraxia of speech, similar to the nonfluent variant primary progressive aphasia phenotype (nfvPPA). Another cortical variant is the PSP with frontal presentation (PSP-F), which presents clinical features resembling the behavioral variant of frontotemporal dementia (bvFTD), with intense personality and cognitive disturbances prior to developing motor features. Finally, another cortical variant is the corticobasal variant (PSP-CBS), a phenotype mostly linked to CBD pathology, although it might be found in cases with a PSP neuropathology. In a previous postmortem study, PSP-CBS was present in only six of 179 PSP pathological cases^5^. Another rare and somewhat controversial phenotype not included in the MDS PSP criteria is the PSP-cerebellar ataxia (PSP-C), in which cerebellar ataxia is observed before the typical motor findings. This variant was reported in two previous autopsy studies, albeit rarely found among PSP pathological cases^18^ ^,^ ^22^. 

 Regarding CBD clinical variants, CBS (CBD-CBS) is the clinical presentation in approximately 25-50% of autopsy-confirmed cases^5^ ^,^ ^23^ ^,^ ^24^. Concerning classical motor and cortical CBD features, there is notable asymmetry. It is usually characterized by akinetic-rigid parkinsonism, dystonia, and myoclonus, associated with cortical symptoms such as ideomotor apraxia, alien limb phenomena, aphasia, or cortical sensory deficits. There are many available criteria for CBS, and they differ considerably^25^ ^,^ ^26^. In the current criteria, probable CBS is characterized by an asymmetric presentation with at least two extrapyramidal dysfunctions of limb rigidity/akinesia, limb dystonia, and limb myoclonus, plus two cortical dysfunctions of orobuccal or limb apraxia, cortical sensory deficits, and alien limb phenomena^25^. Usually, symptoms often start in one limb, which is commonly described as rigid or “clumsy”. Such rigidity is intense and of mixed nature, with aspects of rigidity, paratonia, and dystonia, associated with marked bradykinesia and commonly the most prevalent motor symptom. The typical scenario is a progressive rigidity and apraxia in one upper limb, then involvement of either the ipsilateral lower limb or the contralateral upper limb, eventually leading to severe disability several years later. Noteworthily, CBS is a clinical syndrome, not a designed pathology, and has many possible pathologies beyond CBD, including Alzheimer’s disease (AD), PSP, Pick’s disease, and others^23^. 

 Another well-described CBD phenotype is CBD-Richardson (CBD-RS) when patients develop abnormal eye movements and symmetrical parkinsonism similar to those seen in patients with PSP^27^ ^-^ ^29^. A previous study highlighted subtle differences in eye movement disorders in patients with CBD-RS compared to PSP-RS, such as increased saccadic latencies with preserved velocity seen in patients with CBD^5^. 

Other CBD variants are predominantly cognitive syndromes. They might show a phenotype similar to bvFTD (CBD-bvFTD) or visuospatial dysfunctions resembling the posterior cortical atrophy syndrome (CBD-PCA). The latest CBD diagnostic criteria grouped these cognitive variants into a frontal behavioral-spatial syndrome (CBD-FBS)^25^. CBD pathology also can manifest as nonfluent PPA and primary progressive apraxia of speech (PPAOS), named CBD-nfvPPA variant. A prior study suggested that patients with PSP pathology had more PPAOS without nfvPPA, while patients with CBD pathology showed both PPA and PPAOS^30^. Noteworthily, although PSP and CBD usually demonstrate some preferential clinical manifestations, the width of their clinical manifestations encompasses the same heterogeneous spectrum^31^. 

PSP and CBD are mainly sporadic diseases; however, rare familial forms have been described. More than 50 mutations in the microtubule-associated protein tau (MAPT) gene have been identified^32^, and some of them can result in clinical phenotypes and pathological features equal to PSP and CBD. Moreover, they share similar genetic risk factors, such as the MAPT H1 haplotype, APOE e2/e2, and single nucleotide polymorphism in the MOBP gene^33^. 

## PSP AND CBD DIAGNOSTIC CRITERIA

Clinical diagnostic criteria were recently redefined for PSP^34^ and CBD^25^. However, the definitive diagnosis of PSP and CBD is still only established at *postmortem* examination.

The MDS-PSP criteria^34^ recognized suggestive forms of PSP and operationalized diagnoses of non-RS phenotypes. There were 12 core clinical features in four clinical domains: ocular motor dysfunction (O), postural instability (P), akinesia (A), and cognitive dysfunction (C). Each clinical domain includes three features graded from the more to the least specific for PSP diagnosis. The criteria also included clinical clues and imaging findings. They proposed three degrees of diagnostic certainty: probable, possible, and suggestive of PSP, and the predominant clinical variant^34^. A diagnosis of probable PSP requires vertical gaze palsy or slow vertical saccades, with one more core clinical feature. Conversely, the last criteria from the National Institute for Neurological Disorders and Society for PSP (NINDS-SPSP)^35^ only considered the PSP-RS phenotype. Previous clinicopathologic studies have shown a better sensitivity for the MDS PSP criteria than for NINDS-SPSP criteria^36^. 

Before the latest CBD diagnostic criteria, others have been proposed. However, they demonstrated low sensitivity and specificity, reflecting only the CBS phenotype^37^. In light of the expanding understanding of CBD clinicopathologic correlations, a specialist consensus with brain bank cases and a critical literature review developed new diagnostic criteria for CBD and CBS^25^.

The current CBD diagnostic criteria are the first to incorporate phenotypes other than CBS into the CBD clinical continuum. They proposed two diagnostic classifications for CBD: (1) “clinical research criteria for probable sporadic CBD” (cr-CBD) and (2) “possible CBD” (p-CBD). In the most specific one, namely cr-CBD, age must be greater than or equal to 50 years, and there should not be a family history. Included phenotypes were probable CBS, nfvPPA, and frontal behavioral-spatial syndrome. Furthermore, these last two phenotypes must contain CBS clinical components. The p-CBD criteria aimed to be less restrictive with higher sensitivity. There was no minimum age, and positive family history was allowed. In addition, other phenotypes such as possible CBS and PSP phenotypes were included. In both scenarios, the progression must be gradual with insidious onset and a minimum duration of one year^25^. 

Despite recent efforts to refine CBD clinical criteria, validation studies with clinicopathological cohorts demonstrated that there is still poor sensitivity within two years of disease onset, and patients without CBD pathology can fulfill the cr-CBD clinical criteria. Moreover, there is low specificity for distinguishing CBS due to underlying CBD pathology from others, such as Alzheimer’s disease^38^ ^,^ ^39^. 

The MDS PSP criteria also proposed the novel diagnostic category “probable 4R-tauopathy” to address the phenotypic clinical overlap between PSP and CBD, thus improving their *antemortem* clinical recognition^40^. These diagnostic criteria were introduced to recognize patients with clinical syndromes predicting the underlying four-repeat tauopathy pathology with high specificity, although only moderately specific for PSP pathology^40^. They comprise all “probable PSP”^34^, possible PSP-SL, and possible PSP-CBS. These novel criteria were highly specific in a previous *postmortem* validation study^40^, and the clinical concept of 4R-tauopathies might have advantages for clinical practice and research. 

## PSP AND CBD PATHOLOGY ASPECTS

PSP and CBD belong to a group of diseases called tauopathies characterized by abnormal deposition of hyperphosphorylated tau protein in the brain. In contrast to Alzheimer’s disease, tau pathology is the main driver of neurodegeneration in PSP and CBD. In the brains of PSP and CBD patients, tau pathology is observed in neurons and glial cells and is predominantly comprised of four-repeat tau isoforms^10^ ^,^ ^41^. Tau protein is the major neuronal microtubule-associated protein (MAP) encoding by the microtubule-associated protein gene (MAPT) and promotes the assembly and stabilization of microtubules. The alternative splicing of the exons 2,3 and 10 generates six tau isoforms with four and three microtubule-binding domains (4R- and 3R-tau, respectively). An equal proportion of 3R and 4R isoforms are observed in normal human brains^42^. 

 The mechanism by which tau becomes nonfunctional is not entirely understood, but post-translational modifications may play an important role. Hyperphosphorylation is the most important and impacts microtubules' stability and axonal transport. The decreasing tubulin-binding capacity impairs the interaction between tau and microtubules, leading to microtubule disorganization with protein self-polymerization and aggregation^42^ ^,^ ^43^. 

**Figure 1.**
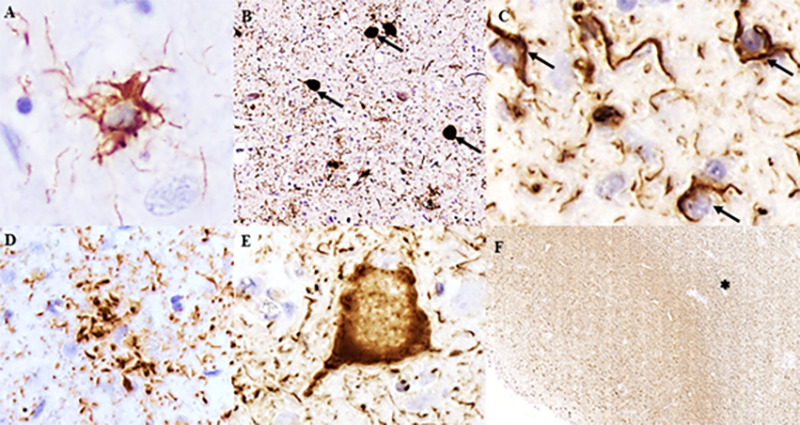
Tau pathology in PSP and CBD. Immunostaining for hyperphosphorylated tau antibody (CP13) shows (A) tufted astrocyte and (B) globose tangles (arrows) found in PSP; (C) oligodendroglial coiled bodies observed in both PSP and CBD with different degrees and localization; (D) astrocytic plaque, a hallmark of CBD; (E) ballooned neuron frequently observed in cortical areas in CBD; (F) severe involvement of white matter (*) in the inferior temporal gyrus in CBD case. Scale bars: A, D, E-50μm; B-200μm; C-20μm; F- 500μm.

According to the distribution and burden of neuronal and glial tau pathology and the involvement of different brain areas, the neuropathological phenotypes of PSP and CBD can be distinguished. Atrophy in the subthalamic nucleus, in the brainstem tegmentum, and depigmentation of substantia nigra are neuropathological features of PSP. Microscopically, a high density of globose neurofibrillary tangles, the most characteristic neuronal lesion, in basal ganglia and brainstem are found in typical PSP cases (Figure 1). However, diffuse granular cytoplasmic immunoreactivity in neurons can be present. In addition, neuropil threads are observed in cortical and subcortical areas, mainly in the striatum, globus pallidus, substantia nigra, and subthalamic nucleus^41^. Glial inclusions are located in white and gray matter in variable amounts and distributions. Tufted-astrocytes (Figure 1) are not pathognomonic of PSP but are the most disease-specific pathological finding, mainly observed in the precentral gyrus, striatum, and superior colliculus^41^. Oligodendroglial inclusions in the form of coiled bodies (Figure 1) are numerous in white matter tracts in the basal ganglia, thalamus, and brainstem. 

Asymmetric focal cortical atrophy in the superior frontal and parietal regions and depigmentation of the *substantia nigra* can be observed in CBD cases. In contrast with PSP, CBD shows more prominent neuronal tau pathology in the forebrain. Numerous neuropil threads are found in white and gray matter associated with variable amounts of coiled bodies, and astrocytic plaques, mainly in affected cortical areas and striatum, are hallmarks of CBD (Figure 1). The burden of coiled bodies is lower compared to PSP. Neuronal tau pathology differs from PSP by the presence of achromatic ballooned neurons in affected cortical areas and small globose tangles, and coiled bodies in *substantia nigra* and locus coeruleus (Figure 1). Besides the type of astroglial tau pathology, the severe involvement of the white matter in CBD and the presence of neurofibrillary tangles in subcortical areas in PSP are some neuropathological findings that can help distinguish PSP and CBD (Figure 1). All neuropathological lesions observed in PSP and CBD are immunoreactive for 4R-tau antibodies but negative for 3R-tau^41^. 

## NEUROIMAGING AND BIOFLUIDS BIOMARKERS

There is growing interest in developing disease-specific biomarkers to aid in predicting pathology in the *antemortem* diagnosis of neurodegenerative diseases. Tau disease pathology-targeted therapies are currently being developed in clinical trials^44^. Hence, there is a need for diagnostic biomarkers to detect PSP and CBD pathology in presymptomatic individuals or early disease phases. Moreover, essential insights into the pathophysiological mechanisms and clinical symptoms have been gained by using advanced neuroimaging techniques in PSP and CBD.

The main biomarkers under study regarding PSP and CBD/CBS include structural imaging modalities such as magnetic resonance imaging (MRI), molecular imaging with functional imaging and specific ligands using positron emission tomography (PET), single-photon emission tomography (SPECT), and biofluid biomarkers such as cerebrospinal fluids (CSF) and serum components. 

### MRI

**Figure 2.**
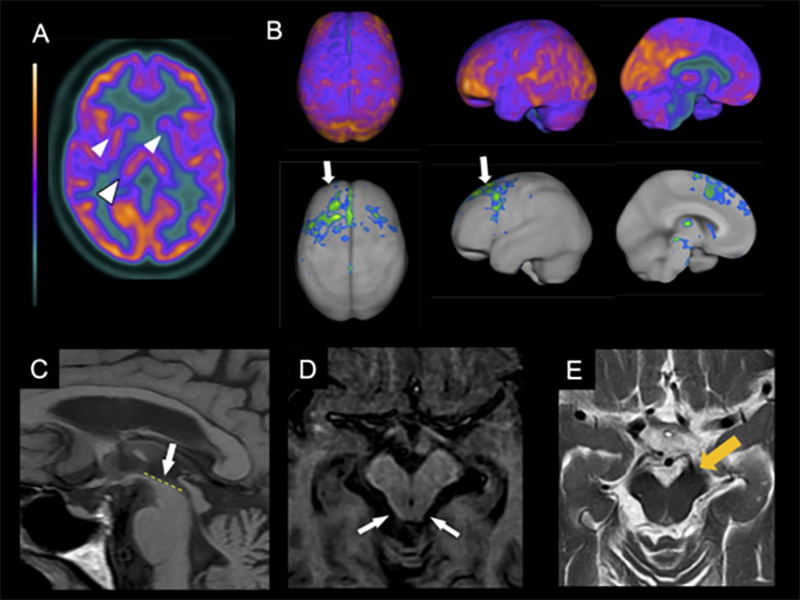
Neuroimaging features of PSP. (A) FDG-PET axial images show a mild bilateral reduction of the metabolism in the thalamus and less evidently on basal ganglia, mainly in the caudate (white arrowheads). (B) FDG-PET 3D-stereotactic surface projection (3D-SSP, software Cortex ID suite, GE Healthcare) shows moderate hypometabolism on both frontal lobes, including the dorsomedial and dorsolateral prefrontal cortices and medial frontal areas, with an extension to the middle cingulate gyri and paracentral areas in the medial projection (white arrows). Upper row - regional glucose metabolism projection; lower row Z-score map of the FDG-PET images highlighting the areas with metabolic impairment compared to a control group paired by age (Z score threshold of -2.0 SD). (C) T1w MRI in the medial projection shows a flattening in the outline of the superior aspect of the midbrain (C - white arrow). Axial images show a reduction of the anteroposterior midline midbrain diameter on T1w (D) and T2w (E) MRI (white arrows on D, orange arrow on E).

In PSP, the imaging hallmark is midbrain atrophy, which can be assessed through the visual identification of the “hummingbird sign”^45^ (specificity 99%, sensitivity 50%) on the sagittal plane, the “morning glory flower sign”^46^ (specificity 97%, sensitivity 37%) on the axial plane, and the superior cerebellar peduncle atrophy in the coronal plane (Figure 2)^47^ ^,^ ^48^. More objective measures that can help diagnose PSP include the area, diameter, and volume of the midbrain^49^, pons-midbrain area ratio^50^, and the parkinsonism index MRPI (magnetic resonance parkinsonism index), calculated through the measurement of the ratios of the pons to midbrain area and middle cerebellar peduncle to superior cerebellar peduncle widths^51^. The latter is especially sensitive and specific for distinguishing PSP from PD, multiple system atrophy-parkinsonian type (MSA-P), and healthy controls^51^. Apparent diffusion coefficient (ADC) increased values in the putamen and superior cerebellar peduncle have good sensitivity and specificity in differentiating PSP-RS from PD^52^. Also, diffusion tensor imaging (DTI) may show a degeneration pattern suggestive of PSP-RS^53^. 

Although these MRI findings are valuable for differentiating PSP-RS from other parkinsonian syndromes with high specificity, they are less sensitive than PSP clinical diagnosis in its distinction from other degenerative parkinsonisms^46^ ^,^ ^49^. Another limitation is that most studies restricted their subjects to patients presenting with a PSP-RS phenotype. One of the few studies that included PSP presentations other than RS showed that cortical variants (PSP-CBS, PSP-F, PSP-SL) had more frontal atrophy than subcortical variants (PSP-RS, PSP-P, PSP-PGF)^54^. 

Resting-state functional MRI in PSP revealed a perturbation of extensive neural networks, especially connections towards the dorsal midbrain tegmentum^55^, as well as widespread disruption of cortical-subcortical connectivity^56^. Frontostriatal hypoactivity can also be seen in functional MRI during vertical saccades in PSP compared with controls^57^. Another functional MRI study assessing speech tasks in PSP demonstrated strong activation of the lingual gyrus and reduced activation of the primary areas with the recruitment of remote areas^58^.

**Figure 3.**
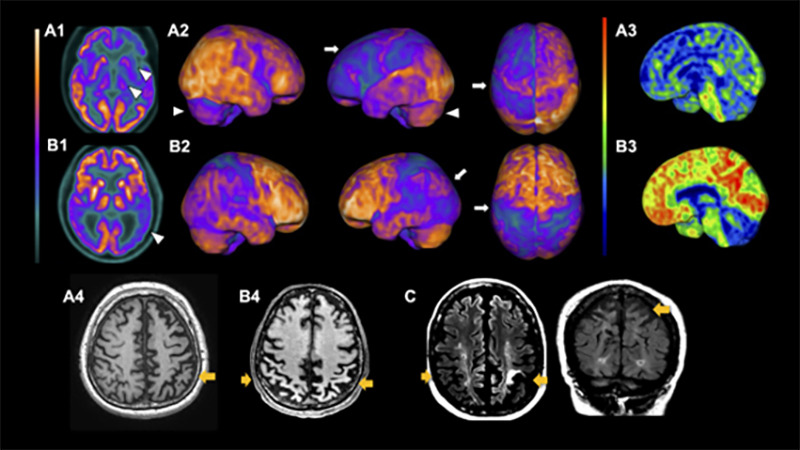
Neuroimaging features of CBD and CBS. Upper row: (A1) FDG-PET axial images of an individual with corticobasal degeneration (CBD) shows the classical asymmetric hypometabolism in the left basal ganglia and thalamus (white arrowheads). Contralateral cerebellar hypometabolism due to diaschisis is seen on FDG-PET 3D-stereotactic surface projection (arrowheads in A2)(3D-SSP, software cortex ID suite, GE Healthcare). (A2) A severe and extensive asymmetric frontoparietal cortical hypometabolism is seen, also in the sensory and motor cortex, worst on the left side, with severe asymmetric impairment of the frontal lobes. (A3) PIB-PET in the left medial projection of the same patient tested negative for amyloid deposition. This pattern is consistent with CBS due to 4R tauopathy (CBD). Middle row: B1) FDG-PET axial images of an individual with corticobasal syndrome due to Alzheimer's Disease (CBS-AD). A posterior bilateral temporoparietal hypometabolism is seen, only slightly asymmetric, without impairment of the basal ganglia, thalami, and cerebellum (white arrowheads). B2) FDG-PET 3D-stereotactic surface projection shows a severe and extensive hypometabolism predominantly in the parietal lobes, however also including the sensory and motor cortex, slightly worse in the left side, with the left side relative sparring of the frontal lobes. (B3) PIB-PET in the left medial projection shows diffuse cortical amyloid deposition. This is the typical pattern related to CBS-AD. Lower rows - atrophy patterns seen on CBS. (A4) T1-weighted MRI with asymmetric frontoparietal atrophy, also worst on the left side (orange arrow) of the same patient on A1,2 and 3. (B4) MRI of the same patient with CBS-AD shown on B1,2 and 3, showing bilateral parietal cortical atrophy (orange arrows). (C) T2/FLAIR MRI shows severe atrophy and asymmetric subcortical parietal gliosis, especially of the perirolandic cortex on the left side, on a patient with late-stage CBD.

CBD, in its turn, usually presents supratentorial patterns of atrophy, mainly asymmetrical patterns in the posterior frontal, superior parietal lobe, and basal ganglia (Figure 3). Cortical thinning and subcortical volume loss prominently involve the hemisphere contralateral to the more affected limb. Also, motor severity negatively correlates with the contralateral cortical thinning in the precentral and postcentral gyri and with volumes of putamen^59^. Moreover, multimodal MRI studies search for CBS patterns of structural lesions that may suggest underlying pathology. A clinicopathologic study suggested that patterns of gray matter loss in CBS differ according to the underlying pathology^60^. Individuals with CBS and a *postmortem* diagnosis of CBD and PSP displayed similar focal atrophy at premotor and supplementary motor areas. In contrast, patients with underlying FTD-TDP43 and AD pathology had a more widespread pattern of gray matter loss at the frontotemporal lobe and temporoparietal regions, respectivelly^60^. 

### PET

Molecular neuroimaging using PET allows for quantitative visualization of functional processes *in vivo.* [^18^F]fluorodeoxyglucose (FDG) is the most commonly used radiotracer for assessing regional brain glucose metabolism as a marker of neuronal function. Additionally, specific-pathology ligands, such as the amyloid-PET and tau tracers, are leading the frontiers of neurodegenerative diseases biomarkers with their role in disclosing underlying pathology. 

The brain metabolic patterns obtained from FDG-PET assist in the early diagnoses of neurodegenerative diseases and is helpful to differentiate Parkinson’s disease from atypical parkinsonism^61^ ^,^ ^62^. FDG-PET in PSP-RS shows a characteristic pattern of hypometabolism in the midbrain, basal ganglia, thalamus, and frontal lobes, including prefrontal, anterior cingulate, premotor, and motor regions^61^ ^,^ ^63^. A typical asymmetrical hypometabolism in CBD involves the frontal and parietal lobes, basal ganglia, and thalamus (Figure 2). 

Although CBD displays a characteristic frontoparietal asymmetric hypometabolism^64^, CBS shows a more complex set of metabolic patterns due to its diverse neuropathologies. A recent study with neuropathologic examination showed that CBS underlying pathologies are associated with different metabolic degeneration patterns and described hypometabolism for CBS-CBD, CBS-AD, and CBS-PSP^65^. Another prospective study using FDG-PET and amyloid-PET in a CBS cohort showed that individual brain metabolic patterns could distinguish with high specificity and accuracy CBS due to AD pathology from CBS non-AD pathological variants^66^, suggesting that it might be routinely used in the clinical workup of CBS. Accordingly, CBS-AD shows distinct metabolic signatures, such as a more posterior temporoparietal pattern and less frontal hypometabolism, compared to CBS not related to AD pathology^65^ ^,^ ^66^. As neurodegeneration modulates metabolism, the metabolism also can depict its clinical features and clinical variants in CBS^66^ ^,^ ^67^. 

Concerning PSP, however, there is still uncertainty regarding its usefulness in distinguishing PSP clinical variants. A previous study demonstrated that FDG-PET is a reproducible diagnostic tool for PSP-RS and PSP-P variants^68^.

Moreover, PET tracers that bind to the protein tau aggregated as neurofibrillary tangles have been developed and performed in PSP and CBD patients. Prior studies using the first generation of tau-targeting tracers, the most widely used ^18^F-AV-1451 (also known as flortaucipir), showed good correspondence between *in vivo* imaging and *postmortem* PSP and CBD evaluation^54^ ^,^ ^69^ ^,^ ^70^. Also, it was helpful to distinguish between CBS, PSP, and AD^69^, and the uptake in globus pallidus might be a valuable measure for differentiating PSP-RS from PD^70^. Nevertheless, as the ultrastructural characteristics of tau filaments differ across diseases, first-generation tau tracers demonstrate more affinity to paired helical filaments found in AD (tau 3R/4R) than straight filaments found in 4R-tauopathies^10^. 

In contrast, second-generation tau PET tracers such as [^18^F]PI-2620 demonstrated more specificity for 4R-tauopathies. Elevated uptake has been observed in several areas in PSP-RS, such as globus pallidus, subthalamic nucleus, putamen, substantia nigra, and dentate^71^. The same tracer has been studied in CBS and demonstrated to help diagnose underlying pathology and potentially monitor disease progression^72^.

 The first specific tracer to amyloid-beta applied in human studies was the [^11^C]Pittsburgh Compound-B (PIB). Later, the second generation of amyloid tracers was developed and named florbetapir, flutemetamol, and florbetaben. Noteworthily, amyloid-PET interpretation has some limitations as the fact that it is positive in about 20-30% of cognitively normal individuals and non-AD dementias, especially when older (mostly above 70 years old)^73^. Currently, amyloid-PET is available for clinical use and is approved by many regulatory agencies worldwide. In CBS, it can be a valuable tool to distinguish cases related to underlying AD pathology from cases related to CBD or PSP. A prior study that used amyloid-PET in CBS patients showed that, among 14 patients, four were positive with high PIB binding (a standardized uptake ratio >1.5), indicating underlying AD pathology^74^. Subtle differences in the clinical presentation were noted between groups, with greater impairment of visuospatial function, more frequent deficits in sentence repetition, and more significant functional decline in PIB-positive patients^74^. In a more recent cohort, among 30 CBS patients, 13 had a positive PIB-PET, and they also demonstrated worse cognitive performances^66^ ^,^ ^67^. 

### Fluid biomarkers

Several fluid biomarkers have been assessed in neurodegenerative diseases. It is now established that the increase of total tau (T-tau) and phosphorylated tau (p-tau) with low levels of the 42 aminoacids beta-amyloid isoform (Aß42) are highly sensitive and specific for predicting AD pathology^75^. In contrast, there is not yet an accurate set of fluid biomarkers for differentiating underlying pathologies for parkinsonian disorders. Some studies demonstrated that the neurofilament light chain protein (NfL) is increased in patients with atypical parkinsonian syndromes and may help differentiate them from Parkinson’s disease^76^. 

A previous study assessing 160 AD, PD, CBS, PSP, FTD, and MSA patients and 30 controls tested nine potential CSF biomarkers: T-tau, p-tau, Aß42, NfL, ?-synuclein, YKL-40, MCP-1, and soluble amyloid precursor protein α and β (sAPPα and sAPPβ)^77^. In this study, NfL, ?-synuclein, and sAPP? were the ones that better discriminated PD from atypical parkinsonism, but no biomarkers combination could discriminate between atypical parkinsonism subtypes (PSP, CBD, MSA).

NfL, despite its limited role in differential diagnosis, appears to be helpful as a neuronal lesion marker and to predict disease progression^78^. Several longitudinal studies assessing biomarkers and clinical progression in PSP showed that NfL increases with time and correlates with disease progression^78^.

CSF NfL may increase up to 30% in a one-year interval in PSP patients^79^, and NfL increase accompanies disease severity (as measured by Hoen and Yahr and PSP rating scale) and the decrease of superior cerebellar peduncles’ volume^77^. Serum NfL correlates with CSF NfL levels and may predict worse outcomes at higher levels^79^. NfL has been included in recent clinical trials as part of exploratory endpoints and is promising as a surrogate outcome for future trials^80^. 

In conclusion, in recent years, several prospective or clinicopathologic studies have assessed clinical, radiological, and fluid biomarkers that have helped better understand the heterogeneity and complexity of 4R-tauopathies. Currently, it is acknowledged that their heterogeneous clinical phenotypes are led by pathology distribution and affected neural networks that comprise a primary motor, extrapyramidal, cognitive, and behavioral pathways. Although several diagnosing methods are useful in clinical practice, more studies are needed so the clinician may depend less on pathological findings to make a definitive diagnosis of these disorders.
